# The Impact of COVID-19 on Hospital Admissions in Croatia

**DOI:** 10.3389/fpubh.2021.720948

**Published:** 2021-09-09

**Authors:** Karolina Kalanj, Ric Marshall, Karl Karol, Mirjana Kujundžić Tiljak, Stjepan Orešković

**Affiliations:** ^1^Department of Medical Oncology, Clinic of Oncology, Clinical Hospital Center, Zagreb, Croatia; ^2^Epidemiologist and Independent Consultant in Health System Funding Models, Eaglehawk Neck, TAS, Australia; ^3^Independent Consultant, Melbourne, VIC, Australia; ^4^Andrija Štampar School of Public Health, University of Zagreb School of Medicine, Zagreb, Croatia

**Keywords:** COVID-19, pandemic, health system response, hospital admissions, AR-DRG, data transparency

## Abstract

**Background:** The COVID-19 pandemic disrupted hospital care, as hospitals had to deal with a highly infectious virus, while at the same time continuing to fulfill the ongoing health service needs of their communities. This study examines the direct effects of COVID-19 on the delivery of inpatient care in Croatia.

**Materials and Methods:** The research is a retrospective, comparative analysis of the hospital admission rate across all Diagnosis Related Group (DRG) classes before and during the pandemic. It is based on DRG data from all non-specialized acute hospitals in Croatia, which account for 96% of national inpatient activity. The study also used COVID-19 data from the Croatian Institute of Public Health (CIPH).

**Results:** The results show a 21% decrease in the total number of admissions [incident rate ratio (IRR) 0.8, *p* < 0.0001] across the hospital network during the pandemic in 2020, with the greatest drop occurring in April, when admissions plunged by 51%. The decrease in activity occurred in non-elective DRG classes such as cancers, stroke, major chest procedures, heart failure, and renal failure. Coinciding with this reduction however, there was a 37% increase (IRR 1.39, *p* < 0.0001) in case activity across six COVID-19 related DRG classes.

**Conclusions:** The reduction in hospital inpatient activity during 2020, can be attributed to a number of factors such as lock-downs and quarantining, reorganization of hospital operations, the rationing of the medical workforce, and the reluctance of people to seek hospital care. Further research is needed to examine the consequences of disruption to hospital care in Croatia. Our recommendation is to invest multidisciplinary effort in reviewing response procedures to emergencies such as COVID-19 with the aim of minimizing their impact on other, and equally important community health care needs.

## Introduction

The World Health Organization (WHO) declared the novel coronavirus (COVID-19) outbreak a Public Health Emergency of International Concern on 30 January 2020, and a Pandemic on 11 March 2020 (1). The spread of the virus caused disorder in health systems across the globe as countries attempted to deal with the contagion while at the same time maintaining the integrity of their health systems. Hospitals came under pressure to meet the ongoing health service needs of the community while responding to the additional COVID-19 case load and readjusted their care priorities. The Croatian response to COVID-19 essentially followed measures adopted by other European countries that included closing borders, limiting social interaction and creating COVID-19 isolation wards within hospitals (2).

As of the end of April 2021, Croatia reported some 332,183 COVID-19 cases and 1,997 COVID-19 caused deaths per million people, which is near the median point for European countries (3). The first COVID-19 case in Croatia was diagnosed on 25 February 2020. Three weeks later, and in the face of an increasing COVID-19 patient load and growing risk of contagion, the Croatian Government took steps to adapt hospital care delivery to the perceived needs of the pandemic. In Zagreb, the national capital, three hospitals were designated as COVID-19 centers and patients with COVID-19 related conditions requiring hospital care were admitted to those facilities. Four similar centers were established in the regions and in addition, most major hospitals established COVID-19 isolation wards. Under this reorganization, 15% of hospital beds in Croatia were designated as COVID-19 beds (3,599 beds out of a total of 23,597) (4).

Concurrently, hospital staffing schedules were reorganized, and the hospital workforce was divided into two groups, with each working in 2-week shifts. The aim of this strategy was to ensure that backup staff were available to replace potentially infected and COVID-19 positive workers. This workforce management measure was in place for a period of 6 weeks and ended at the beginning of May, once the perceived risk of COVID-19 infections among hospital staff diminished.

Under revised admission procedures, only patients who tested negative for COVID-19 were admitted to general wards for non-COVID-19 related conditions. Patients who needed immediate acute care and proved to be COVID-19 positive, were treated within COVID-19 isolation wards along with other patients who required hospital care due to COVID-19 infection. The realignment of hospital care delivery and reprioritization of needs resulted in the general post-ponement of elective procedures as priority was given to the treatment of COVID-19 admitted patients and urgent non-COVID-19 cases.

The introduction of shift work in hospitals also resulted in a reduction in hospital outpatient consulting hours which most likely had an impact on the admission rate, given that non-emergency admissions in Croatia are initiated by hospital-based specialists in an outpatient setting.

The aim of the study is to assess the direct effects of COVID-19 on inpatient care delivery in Croatia, to identify which types of cases were the most affected and to examine the potential reasons for such outcomes.

## Methods

### Study Design and Data Sources

Information sources for the study are publicly available data from the Croatian Health Insurance Fund (CHIF) and the Croatian Institute of Public Health (CIPH). The CHIF data set comprised inpatient data grouped in accordance with Australian Refined Diagnosis Related Groups (AR-DRGs), for the period 1 January 2017 to 31 December 2020 (Supplementary Table 1) (5). The Croatian DRG system is based on a variant of the Australian AR-DRG system, utilizing a combination of the ICD-10AM and ICD-10 classifications for the coding of diagnosis, and Australian Classifications of Health Interventions (ACHI) for the coding of procedures. The DRG grouping algorithm is based on AR-DRG version 5.2 which assigns cases to 671 DRG classes (6).

The study examined data from all non-specialized acute hospitals in Croatia that serve a population of 4.2 million people, accounting for 96% of inpatient activity. The observed hospital network comprised 11 tertiary level hospitals and 22 secondary level hospitals.

The analysis compared the number of cases recorded in each DRG class. It focused on those DRG classes that were driven by either principal diagnoses or procedures that may have signaled admissions due to COVID-19, as well as those classes related to conditions such as cancers, cardiac and respiratory disease, and stroke.

The coding of principal diagnoses for cases with COVID-19 respiratory manifestations in Croatia was based on the International Classification of Diseases (ICD10 codes)^1^ which resulted in DRG class grouping as either E62A, E62B or E62C (Respiratory Infections/Inflammatory Conditions). Otherwise, if COVID-19 patients required (invasive) ventilatory support the episode was grouped as either A06Z or E40Z (Respiratory System Diagnosis & Ventilator Support). If the case involved high flow oxygen therapy, it was classified as belonging to the E41Z (Respiratory System Diagnosis and Non-Invasive Ventilation) DRG class.

For the purpose of analyzing the effect of the COVID-19 pandemic on non-COVID-19 cases, we used DRG data reported in 23 Major Diagnostic Categories (MDC) which represent the grouping of patients based on their principal diagnoses which, according to DRG coding practice, is generally the main reason for admission (7). Each MDC corresponds to a single body system or etiology, and the system is harmonized with the ICD10 classification structure.

The study also utilized the COVID-19 data set provided by the CIPH and which incorporated information on reported national incidence of the COVID-19 virus, including the total number of COVID-19 related hospital admissions and the number of patients on ventilators (Supplementary Table 2) (8). The CIPH classified COVID-19 admissions as those cases that have a positive polymerase chain reaction test for severe acute respiratory syndrome coronavirus 2 (SARS-CoV-2). It used WHO's COVID-19 coding guidelines as the standard for COVID-19 reporting by Croatian hospitals, and all admitted COVID-19 positive cases were identified by ICD10 code U07.1 (Covid-19, virus identified) as the secondary diagnosis.

Our study did not require informed consent nor ethical approval given that the data used were completely anonymized and publicly available from CHIF and the CIPH in compliance with Croatian data protection regulations.

### Data and Statistical Analysis

As the first step of the data analysis we calculated changes in hospital admissions and compared the average number of acute inpatient cases over a 3-year period (2017–2019) with the number of cases over a 12-month period in 2020. We used DRG data grouped in MDCs in order to determine the extent to which, and for which conditions the onset of the pandemic altered the pre-COVID-19 casemix across the hospital network.

The next step of the analysis involved calculating changes to hospital admissions for six COVID-19 related DRG classes. Thereafter, we calculated changes in inpatient activity in non-COVID-19 related DRG classes for case groups that included cancer, cardiovascular diseases and stroke.

For each of the two time periods (2017–2019 and 2020) the incidence rate ratio for all MDCs, COVID-19 and cancer related DRGs classes was calculated as a number of events (inpatient admissions) divided by the total population during the two respective time periods (based on the Croatian Bureau of Statistics population estimates for 2017-2021).^2^

The IRR was estimated as a ratio of the incidence rate for 2020 to that for 2017–2019. The 95% confidence interval limits for the IRR were estimated using the Wald method. Additionally, *p*-values of a Pearson chi-square test for the hypothesis of IRR being equal to one (i.e., the hospitalization incidence rate in 2020 being equal to that in 2017–19) were calculated. All statistical analyses were performed using SAS 9.4 with significance level set at *p* < 0.05. (SAS Institute Inc., Cary, NC, USA).

## Results

The CIPH began reporting COVID-19 data on 14 April 2020. In 2020 it recorded 20,609 hospital admissions with a positive polymerase chain reaction test for severe acute respiratory syndrome coronavirus 2 (SARS-CoV-2). For 2020, the total number of admissions compared to average admission for the previous 3 years decreased by 21% (an average of 532,860 cases between 2017 and 2019 to 420,890 cases in 2020). The decline in the number of admissions was similar in both tertiary and secondary level hospitals.

**Figure 1:**
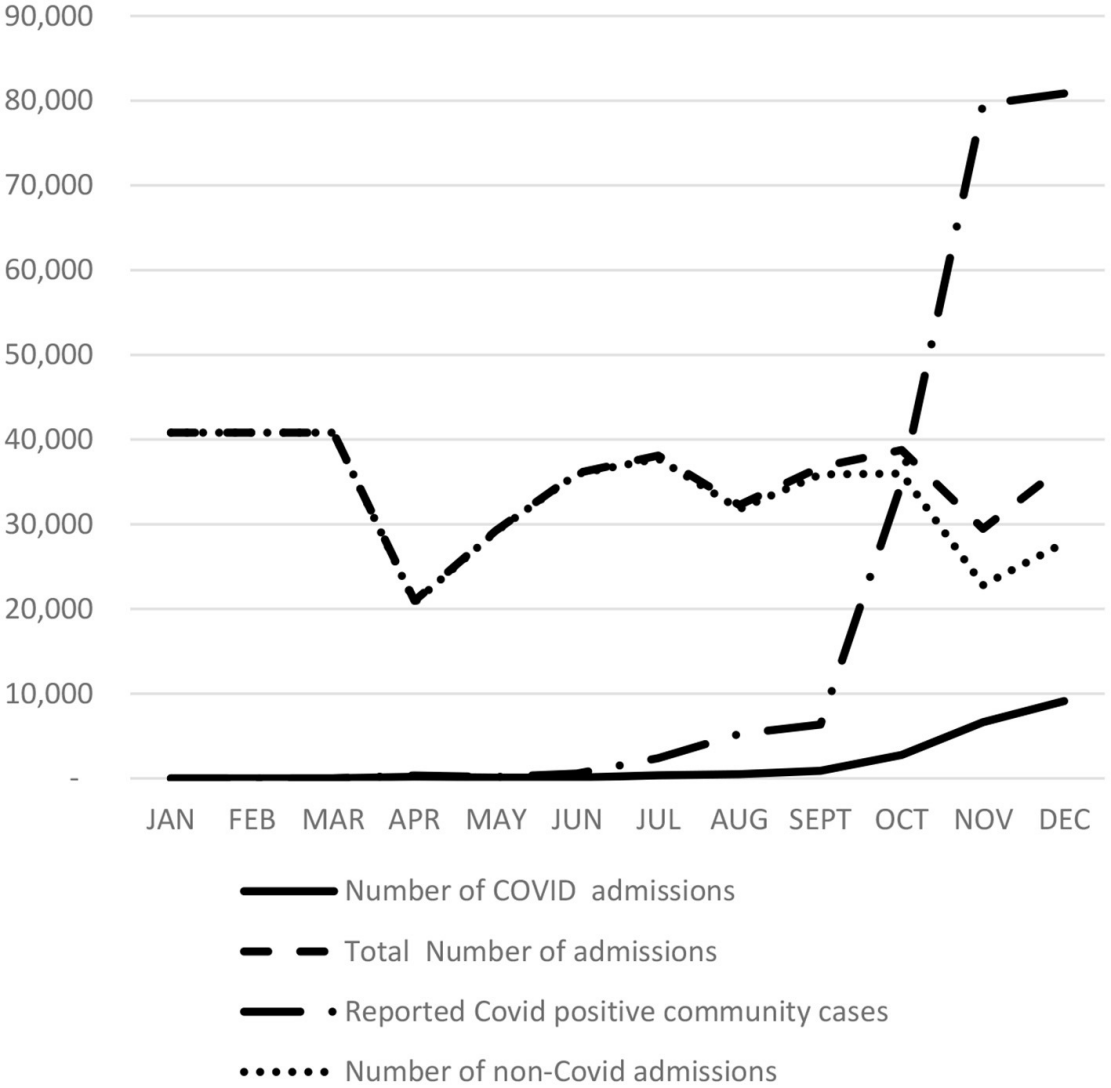
Comparison of Total Monthly Admissions, COVID-19 Related and non-COVID-19 Admissions. Source: Monthly admission data from CHIF—January, February, March data is an average for the first quarter of 2020; Covid-19 admission data from PHI (PHI begun recording Covid-19 data on 14th April, 2020).

Figure 1 shows monthly inpatient case activity for 2020 in the 33 hospitals covered in the study (based on CHIF data), along with the number of reported COVID-19 cases in the community and monthly admissions of patients with a COVID-19 diagnosis (based on CPHI data). The average monthly admission rate over the 3-year pre-COVID period was 44,400 cases, and it stood at an average of 40,810 cases for the first 3 months of 2020 (monthly DRG data was not available for the first 3 months of 2020). The first significant impact of COVID-19 on DRG activity was felt in April 2020 when admissions plunged by ~51% to 20,963 cases. This was the lowest monthly level of acute inpatient activity for 2020. Monthly admissions increased to 36,022 cases in June and oscillated at this level for the remainder of the year.

According to CIPH data, the COVID-19 hospital admission rate was relatively modest until September when the incidence of COVID-19 in the community begun to spike. The number of COVID-19 admissions grew dramatically from 888 cases in September to 9,120 cases in December 2020. During the same period, non-COVID related monthly case activity decreased by 36%, from 35,877 cases in September to a low of 22,822 in November, at a time when the incidence of COVID in the community was peaking.

**Figure 2:**
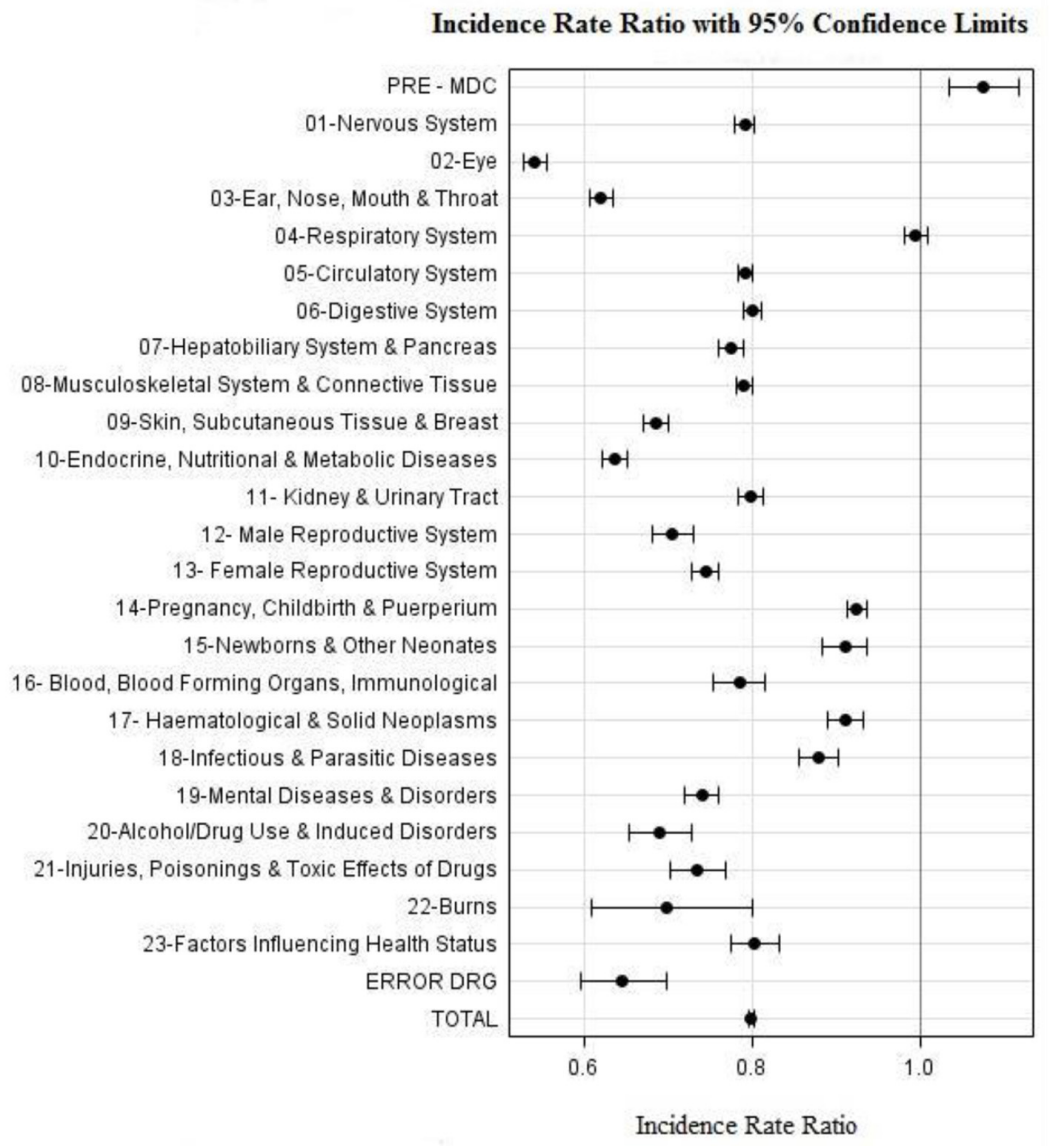
Incidence Rate Ratio per MDC during 2020 as compared to period 2017–2019.

Table 1 compares the average 3-year (2017–2019) pre-COVID DRG case activity to that of 2020 expressed in MDCs. While the average decrease in activity was 21% (*p* < 0.0001), the MDCs in which the decrease in activity was considerably greater than the average included diseases and disorders related to: the Eye by 46% (*p* < 0.0001); Ear Nose and Throat by 39% (*p* < 0.0001); Skin, Subcutaneous Tissue and Breast by 32% (*p* < 0.0001); Endocrine, Nutritional and Metabolic by 37% (*p* < 0.0001); Male Reproductive System by 30% (*p* < 0.0001); Female Reproductive System by 26% (*p* < 0.0001); Mental Diseases and Disorders by 27% (*p* < 0.0001); Alcohol/Drug Use and Induced Disorders by 32% (*p* < 0.0001); Injuries, Poisonings and Toxic Effects of Drugs by 27% (*p* < 0.0001); and Burns by 31% (*p* < 0.0001). Figure 2 shows corresponding IRRs calculated for every MDC.

**Table 1:** Comparison of DRG case activity before, and during Covid 19 in 2020.

**Major diagnostic categories** **(diseases, disorders and other conditions)**	**DRG cases**									***p***
	**2017–19 Average tertiary hospitals**	**2017–19 Average secondary hospitals**	**2017–19 Average total hospitals**	**2020** **Total tertiary hospitals**	**2020** **Total secondary hospitals**	**2020** **All hospitals**	**%** **Change tertiary hospitals**	**%** **Change secondary hospitals**	**%** **Change total hospitals**	
PRE - MDC	3,388	1,544	4,932	3,636	1,609	5,245	7%	4%	6%	0.0003
01-Nervous system	23,241	17,383	40,624	18,056	13,722	31,778	−22%	−21%	−22%	<0.0001
02-Eye	13,223	6,245	19,468	7,329	3,096	10,425	−45%	−50%	−46%	<0.0001
03-Ear, nose, mouth and throat	13,204	7,646	20,850	8,286	4,495	12,781	−37%	−41%	−39%	<0.0001
04-Respiratory system	19,350	19,343	38,693	18,662	19,392	38,054	−4%	0%	−2%	00.4321
05-Circulatory system	38,036	24,537	62,573	30,132	18,885	49,017	−21%	−23%	−22%	<0.0001
06-Digestive system	28,526	22,420	50,946	23,422	16,900	40,322	−18%	−25%	−21%	<0.0001
07-Hepatobiliary system and pancreas	15,687	10,465	26,152	11,324	8,719	20,043	−28%	−17%	−23%	<0.0001
08-Musculoskeletal system and connective tissue	35,314	21,352	56,666	27,404	16,876	44,280	−22%	−21%	−22%	<0.0001
09-Skin, subcutaneous tissue and breast	12,542	7,298	19,840	8,338	5,090	13,428	−34%	−30%	−32%	<0.0001
10-Endocrine, nutritional and metabolic diseases	11,651	4,533	16,185	7,352	2,831	10,183	−37%	−38%	−37%	<0.0001
11-Kidney and urinary tract	14,933	11,697	26,630	11,852	9,183	21,035	−21%	−21%	−21%	<0.0001
12- Male reproductive system	5,093	3,010	8,103	3,719	1,931	5,650	−27%	−36%	−30%	<0.0001
13- Female reproductive system	12,590	7,758	20,348	9,062	5,920	14,982	−28%	−24%	−26%	<0.0001
14-Pregnancy, childbirth and puerperium	28,236	22,864	51,100	25,588	21,132	46,720	−9%	−8%	−9%	<0.0001
15-Newborns and other neonates	5,234	3,854	9,088	4,509	3,672	8,181	−14%	−5%	−10%	<0.0001
16- Blood, blood forming organs, immunological	3,268	2,323	5,591	2,428	1,910	4,338	−26%	−18%	−22%	<0.0001
17- Hematological and solid neoplasms	11,312	3,246	14,558	9,734	3,387	13,121	−14%	4%	−10%	<0.0001
18-Infectious and parasitic diseases	5,726	5,915	11,641	4,745	5,371	10,116	−17%	−9%	−13%	<0.0001
19-Mental diseases and disorders	5,966	6,365	12,331	4,331	4,691	9,022	−27%	−26%	−27%	<0.0001
20-Alcohol/drug use and induced disorders	1,413	1,852	3,265	988	1,237	2,225	−30%	−33%	−32%	<0.0001
21-Injuries, poisonings and toxic effects of drugs	2,398	1,945	4,342	1,712	1,444	3,156	−29%	−26%	−27%	<0.0001
22-Burns	324	174	498	219	125	344	−32%	−28%	−31%	<0.0001
23-Factors influencing health status	5,629	1,181	6,811	4,384	1,022	5,406	−22%	−13%	−21%	<0.0001
ERROR DRG	991	636	1,627	548	490	1,038	−45%	−23%	−36%	<0.0001
**Total**	**317,274**	**215,586**	**532,860**	**247,760**	**173,130**	**420,890**	–**22%**	–**20%**	–**21%**	**<0.0001**

*Source: CHIF and authors calculation*.

MDCs for which the decrease in case activity was less than the average included medical conditions related to: Respiratory System decreasing by 2% (*p* = 0.4321); Pregnancy, Childbirth and Puerperium by 9% (*p* < 0.0001); Newborns and Other Neonates by 10% (*p* < 0.0001); Hematological and Solid Neoplasms by 10% (*p* < .0001); and Infectious and Parasitic Diseases by 13% (*p* < 0.0001). The only MDC experiencing an increase (6%) in activity was Pre-MDC which includes mechanical ventilation DRG (A06Z) episodes which are most likely associated with COVID-19 cases (*p* =0.0003).

**Figure 3:**
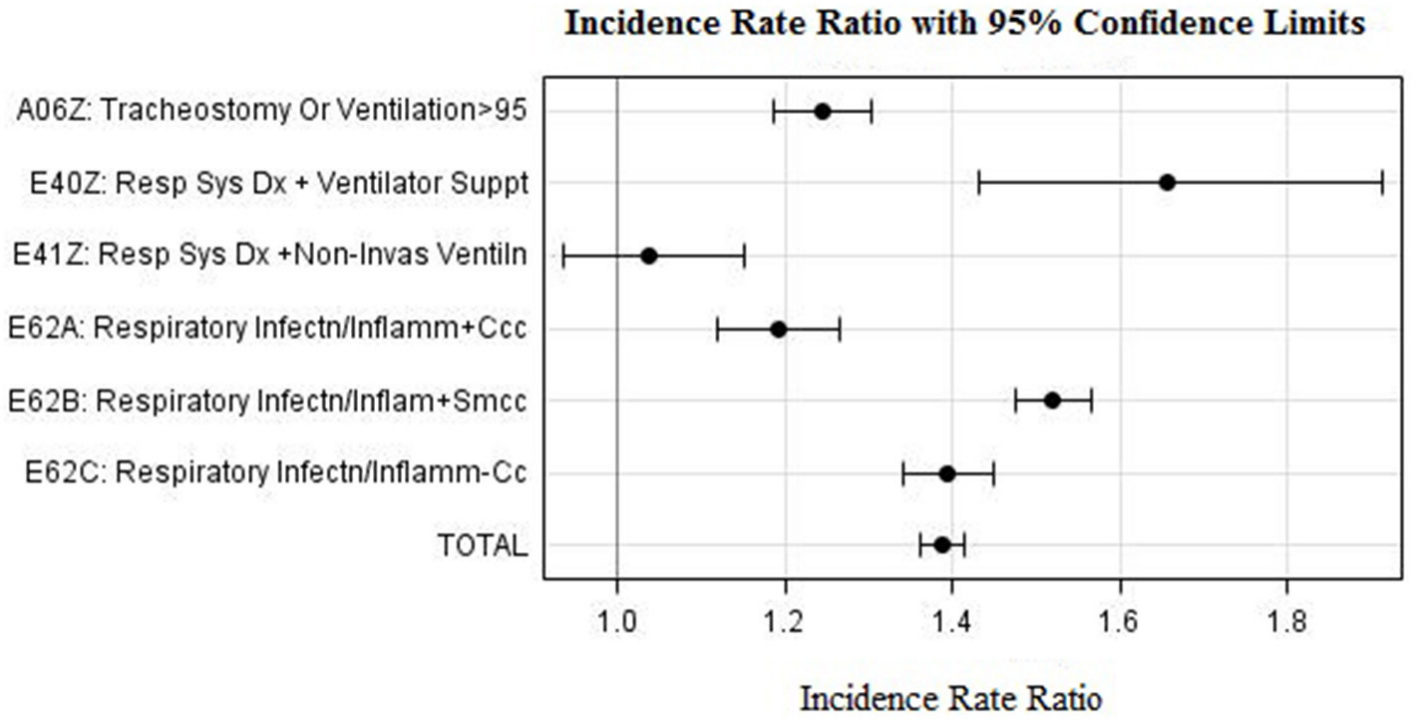
Incidence Rate Ratio for COVID-19 related DRGs during 2020 as compared to period 2017–2019.

Table 2 is based on CHIF data and shows the difference in case activity across six DRG respiratory classes attributable to COVID-19. The data shows an 37% increase in activity in this category, from an average of 17,875 cases over the 3-year pre-COVID period to 24,533 cases in 2020 (*p* < 0.0001). The increase was greater in tertiary level hospitals at 45%, compared to 31% in secondary level hospitals. An exception was DRG class E41Z Respiratory System Diagnosis with Non-Invasive Ventilation for which tertiary hospitals reported a 21% decrease in case activity, while secondary hospitals reported a 112% increase. As shown in Figure 3, we found that IRRs for all six respiratory DRG classes is >1.

**Table 2:** Difference in activity in Covid 19 related DRG classes before, and during Covid 19 in 2020.

**DRG no**	**DRG description**	**DRG cases**									***p***
		**2017–19 Average tertiary hospitals**	**2017–19 Average secondary hospitals**	**2017–19 Average** **of all hospitals**	**2020** **Total tertiary hospitals**	**2020** **Total secondary hospitals**	**2020** **All** **hospitals**	**%** **Change tertiary hospitals**	**%** **Change secondary hospitals**	**%** **Change total hospitals**	
A06Z	Tracheostomy or ventilation > 95	1,951	1,231	3,181	2,424	1,492	3,916	24%	21%	23%	<0.0001
E40Z	Resp sys dx + ventilator suppt	139	155	295	271	212	483	94%	36%	64%	<0.0001
E41Z	Resp sys dx + non-invas ventiln	573	122	695	454	259	713	−21%	112%	3%	0.4925
E62A	Respiratory infectn/inflamm + ccc	765	1,148	1,914	971	1,284	2,255	27%	12%	18%	<0.0001
E62B	Respiratory infectn/inflam + smcc	3,146	4,169	7,315	5,230	5,762	10,992	66%	38%	50%	<0.0001
E62C	Respiratory infectn/inflamm-cc	1,571	2,904	4,475	2,484	3,690	6,174	58%	27%	38%	<0.0001
	**Total**	**8,146**	**9,730**	**17,875**	**11,834**	**12,699**	**24,533**	**45%**	**31%**	**37%**	**<0.0001**

*Source: CHIF and authors calculation*.

**Figure 4:**
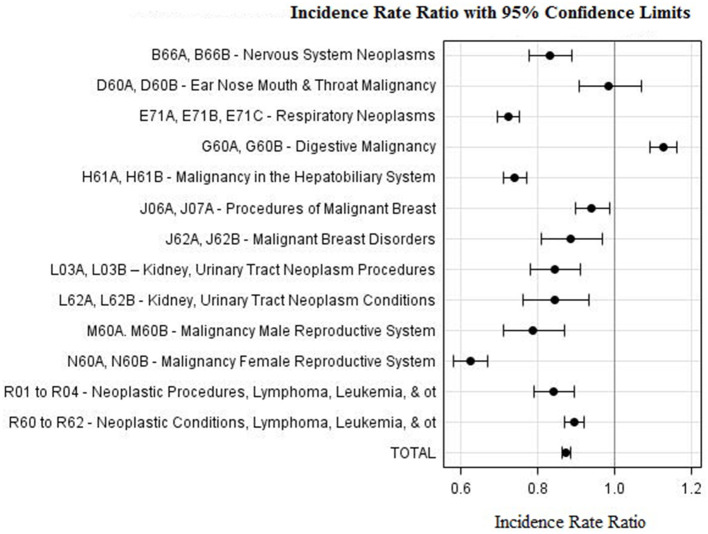
Incidence Rate Ratio Cancer and Neoplasm related DRGs during 2020 as compared to period 2017–2019.

Table 3 shows case activity differences for DRG classes related to the treatment of cancers including DRG classes attributed to both malignancy and neoplastic conditions. Case numbers in these groups decreased by 14%, from an average of 44,206 in the 3 previous years, to 38,176 in 2020 (*p* < 0.0001). Tertiary hospitals experienced a decrease of 16% in cancer and neoplasm related admissions, while secondary level hospitals had a 6% decrease in such admissions. Cancer related DRG classes which experienced greatest reductions in activity were: Respiratory Neoplasms (28%; *p* < 0.0001); Malignancy in the Hepatobiliary System and Pancreas (27%; *p* < 0.0001); Malignancy Male Reproductive System (22%; *p* < 0.0001); and Malignancy Female Reproductive System (38%; *p* < 0.0001). DRG cancer classes in which reduction in activity was below the average included: ENT malignancy (3%; *p* = 0.7115); Procedures of Malignant Breast (7%; *p* = 0.0130); Malignant Breast Disorders (12%; *p* = 0.0076); and Lymphoma and Leukemia (12%; *p* < 0.0001). The only cancer related DRG class in which there was more activity during 2020, was Digestive Malignancy which increased by 11% (*p* < 0.0001). Figure 4 shows IRR values for cancer related DRGs (1 < IRRs < 1) which indicate that oncology case types were affected by the COVID-19 response in different ways.

**Table 3:** Difference in case activity in cancer and neoplasm related DRG classes before, and during Covid 19 in 2020.

**DRG Names**	**DRG cases**									***p***
	**2017–19 Average tertiary hospitals**	**2017–19 Average** **secondary hospitals**	**2017–19 Average** **of all hospitals**	**2020** **Total tertiary hospitals**	**2020** **Total secondary hospitals**	**2020** **All hospitals**	**%** **Change tertiary hospitals**	**%** **Change secondary hospitals**	**%** **Change total hospitals**	
B66A, B66B - nervous system neoplasms	1,113	822	1,936	913	679	1,592	−18%	−17%	−18%	<0.0001
D60A, D60B - ear nose mouth and throat malignancy	907	237	1,144	907	207	1,114	0%	−13%	−3%	0.7115
E71A, E71B, E71C - respiratory neoplasms	4,202	1,840	6,042	2,854	1,467	4,321	−32%	−20%	−28%	<0.0001
G60A, G60B - digestive malignancy	5,728	1,872	7,600	6,405	2,061	8,466	12%	10%	11%	<0.0001
H61A, H61B - malignancy in the hepatobiliary system	4,659	1,195	5,854	2,782	1,499	4,281	−40%	25%	−27%	<0.0001
J06A, J07A - procedures of malignant breast	2,505	914	3,418	2,358	822	3,180	−6%	−10%	−7%	0.0130
J62A, J62B - malignant breast disorders	735	296	1,031	707	196	903	−4%	−34%	−12%	0.0076
L03A, L03B - kidney, urinary tract neoplasm procedures	1,085	295	1,380	932	219	1,151	−14%	−26%	−17%	<0.0001
L62A, L62B - kidney, urinary tract neoplasm conditions	457	383	841	410	291	701	−10%	−24%	−17%	0.0008
M60A. M60B - malignancy male reproductive system	452	433	884	361	326	687	−20%	−25%	−22%	<0.0001
N60A, N60B - malignancy female reproductive system	1,511	540	2,051	817	447	1,264	−46%	−17%	−38%	<0.0001
R01 to R04 - neoplastic procedures, lymphoma, leukemia, and other hematol.	1,530	594	2,124	1,277	487	1,764	−17%	−18%	−17%	<0.0001
R60 to R62 - neoplastic conditions, lymphoma, leukemia, and other hematol.	7,672	2,229	9,901	6,475	2,277	8,752	−16%	2%	−12%	<0.0001
**Total**	**32,556**	**11,650**	**44,206**	**27,198**	**10,978**	**38,176**	–**16%**	–**6%**	–**14%**	**<0.0001**

*Source: CHIF and authors calculation*.

In addition to cancers, activity data indicates decreases in admissions for the following non-elective DRG classes: Stroke (15%); Transitory Ischemic Attack (27%); Major Chest Procedures (31%); Chronic Obstructive Airway (55%); Coronary Bypass (24%); Procedures and Conditions related to Circulatory Disorders (27%); Heart Failure and Shock (13%); Coronary Atherosclerosis (38%); Hypertension (43%); Arrhythmia (26%); Rectal resection (17%); and Renal Failure (15%).

Notably, 2020 saw a 26% decrease in DRG classes attributed to endoscopic diagnostic procedures such as colonoscopies and gastroscopies, compared to the average for the previous 3 years.

## Discussion

### Observations

General admissions in Croatian hospitals in 2020 fell by 112,000 (21%) over the study period despite the additional 20,609 COVID-19 related inpatient admissions reported by the CIPH. An initial dip in admissions occurred in April 2020, soon after the WHO declared COVID-19 a pandemic. In that period, Croatia experienced a 51% drop in hospital admissions, a trend also observed to varying degrees in other countries, across a number of medical specialties and emergency department visits (9–16).

An article by Willan, King et al. published in the British Medical Journal on 20th March 2020 at the onset of the pandemic, reflected that the UK government saw itself on war footing in its fight against the virus. The authors anticipated a need to reorganize hospital departments and to redeploy the hospital workforce in the face of an escalating COVID-19 patient load, while fewer staff are available due to infections. Importantly, they foresaw a situation where such pressures would impact adversely on the established standards of care to the extent that some patients may be harmed due shortcomings in treatment (17).

As the pandemic unfolded however, many countries reported a general decrease in hospital inpatient activity (14, 15, 18, 19) and our study shows that this was also the case in Croatia.

We show that Croatia experienced both a decrease in inpatient activity and decline in non-elective admissions for conditions which incidence is not related to the COVID-19 pandemic, but which otherwise pose a serious health risk if left untreated. In the case of cardiovascular diseases, we calculated a 26% decrease in cases over the study period. By comparison, various studies provide the following results: a United Kingdom (UK) study reported a 58% decrease in cardiovascular cases in March 2020 (9); an Italian study a decrease of 26% (11) during the same period; a US study a decrease of 45% (14); a German study reported a 13–28% decline in interventional treatments for heart failure and cardiac arrhythmias (20); and a multinational study of 12 EU countries showed a 30% reduction in acute coronary syndrome cases during the COVID-19 outbreak (21).

In the case of stroke, our study found that Croatia experienced a 15% decrease in stroke related DRG cases over the study period. In comparison, a UK study reported a 40% reduction in stroke cases over March and April of 2020 (22).

Our study also revealed a disruption in cancer care during the COVID study period. We found a 14% decline in admissions related to cancer and other neoplasms, with the greatest reductions occurring in DRGs attributable to Respiratory Neoplasms (28%) and Malignancy in the Hepatobiliary System (27%). This disruption in cancer treatment was also reported in other countries (23). In Germany, a study reported a 10–20% decrease in cancer related hospital admissions (20) and an Italian study reported a 32% drop in oncology related surgical procedures between March and June 2020 (16). Moreover, a UK study projected that delays in early diagnosis and treatment of cancers will, over a 5-year period, increase cancer mortality rates in the following categories: breast (9%), colorectal (16%), lungs (5%), and esophageal (6%) (24). Another UK study predicted that the cancer mortality rate may increase by 20% over a 12-month period, resulting in ~6,000 additional deaths (25).

Though there does not appear to be a single reason for the general decline in inpatient activity in Croatia, contributing factors are likely to include: disruption within the hospital system given its reorganization to address the perceived requirements of the pandemic; the reluctance of people with healthcare needs to seek hospital care in the face of the perceived threat of acquiring a COVID-19 infection in a hospital setting; hospital staff shortages due to infection and illness among the health workforce; the reprioritization of elective procedures by hospitals; and a decrease in the non-emergency admission referral rate due to the reduction in outpatient hours.

There is however, evidence from international sources to support the argument that patient reluctance to attend hospitals could be a major contributing factor. For example, a number of studies on emergency department (ED) admissions during the COVID-19 pandemic report reductions in both daily attendances and surgical admissions through EDs, indicating that patients may not be presenting for needed care (10, 12, 13, 26). Such patient behavior was investigated in an Italian study (27) which found that 32% of the cohort surveyed, faced delays in scheduled services, 12% refused to attend scheduled services for fear of contagion, 6% avoided health services despite the onset of an acute issue, and 1.5% avoided EDs when in need. Deerberg-Wittram and Knothe propose that avoidance of care by patients in a situation such as the COVID-19 pandemic is an example of Dread Risk, which is a behavioral response in which rare and unexpected events like the pandemic, can trigger irrational risk evading responses such as avoiding hospitals due to the perceived risk of infection, while ignoring the risk that such behavior may result in more serious consequences for the person's health (26). Reichardta et al. provide further evidence of such behavior reporting that German states with increased incidence of COVID-19 experienced a greater decrease in hospital admissions (20).

Though post-poning elective interventions may be acceptable over the short term in order to deal with pressing needs at times of emergencies, such post-ponements are likely to exert pressure on hospitals in the longer term as they endeavor to address growing waitlists. According to data from the UK National Health Service (NHS), the waitlist of people awaiting treatment in England at the end of February 2021 was the highest since NHS records began in 2007 and stood at 4.7 million people, while the number of patients who were waiting more than 52 weeks for routine operations and procedures increased by 73% between December 2020 and February 2021 (28).

In summary, as in many other countries, the instinctive response of health authorities in Croatia to the sudden onset of COVID-19 was to address the perceived priority needs of COVID-19 patients. Analysis of DRG data reveals that, as the pandemic unfolded, this response resulted in a general reduction of hospital inpatient services, including the treatment of non-COVID-19 priority needs. The potential consequences of this drop in inpatient services in Croatia may result in increased mortality rates over the coming years for diseases such as cancer and heart conditions (11, 24).

Future studies using DRG data can reveal to what extent inpatient activity recovers over the coming years from the COVID-19 period. If the findings show, however, that the reduction in activity in certain DRG classes becomes more permanent over time, such studies may also include a re-evaluation of the historic need and clinical necessity of these types of admissions.

### Strengths and Limitations

The underlying strength of this study is the utilization of a full data set on inpatient activity for all non-specialist hospitals in Croatia which account for 96% of all acute impatient activity. It is also the first systematic attempt to describe the impact of SARS-CoV-2 pandemic on acute hospital admissions in Croatia.

The limitations of the study are two-fold. The first is related to the quality of DRG coding and the second concerns the COVID-19 admission data reporting standards.

We expect that the quality of the DRG data in Croatia is adequate for the calculation of inpatient activity given that it is audited by the CHIF and used for hospital payment purposes. Some anomalies may exist however, as in the case where our data shows a 21% decrease in activity in the DRG class E41Z in tertiary level hospitals, whereas secondary hospitals show a 112% increase in activity in the same class. While there may be other reasons for this unexpected result, a potential cause of the difference may be the incorrect coding by secondary hospitals of oxygen therapy as non-invasive ventilatory support. Nonetheless, anomalies such as this, should not have any bearing on the findings of this study.

The matter of COVID-19 admission data reporting standards relates to the question whether cases which were reported as COVID-19 were admitted in order to treat symptoms of COVID-19. CHIF's DRG data shows that there was an increase of 6,658 cases (from the average of 17,875 over the 3-year pre-COVID-19 period, to 24,533 cases in 2020), in the six DRG respiratory classes in which cases could be attributed to treatment for COVID-19. However, this finding does not correspond with CIPH data which reported that Croatian hospitals had 20,609 COVID-19 admissions in 2020. Though the reason for this difference requires further investigation, the variance may be due to the fact that admitted cases reported as COVID-19 by CIPH, may have included admissions for reasons other than respiratory manifestations due to COVID-19. The COVID-19 reporting protocol in use by the CIPH is that every patient seeking hospital care is tested at the point of admission and those who test positive are reported as COVID-19, even if they were asymptomatic.

### Conclusions

The regular and frequent publication of DRG activity data provides opportunities for timely decision making in responding to unfolding emergencies situations such as COVID-19. Moreover, an in-depth analysis of the DRG data set can provide insights into utilization patterns, epidemiology and care outcomes, including mortality rates (29).

Though it appears Croatia has responded comparatively well to the COVID-19 emergency, there is room for improvement. One lesson Croatia can draw from this experience is the need to develop strategies and processes whereby the response to pandemics is not necessarily at the expense of other and equally important community health care needs (30).

One area for improvement is that while the response should be timely, public health authorities need to react proportionally, taking into account the population-wide health risk as the pandemic evolves and inform the public accordingly. The strategy should include an evaluation of the consequences to population health if resources are moved from one care need to another. For hospitals, it would mean that their pandemic response is phased-in as well as possible in line with actual clinical need and organized around specialist task groups with the aim of minimizing disruption to the provision of other services. This approach however, would require the organization of hospitals to become more pliant in their ability to react to changing conditions, and to present as safe patient environments at times of contagion.

In addition, greater use of telemedicine would enhance access to care at a time when distancing measures are in place, and a well-targeted information campaign would educate the public of the deleterious consequences of not seeking care.

## Data Availability Statement

The original contributions presented in the study are included in the article/Supplementary Material, further inquiries can be directed to the corresponding author.

## Author Contributions

KKar, KKal, and SO led study design. KKar and RM were responsible for the statistical analyses. KKal wrote the first draft of the paper. All authors contributed to the development of the research question, study design in relation to the Croatian DRG hospital data, interpretation of the results, critical revision of the manuscript for important intellectual content, and approved the final version of the manuscript.

## Conflict of Interest

The authors declare that the research was conducted in the absence of any commercial or financial relationships that could be construed as a potential conflict of interest.

## Publisher's Note

All claims expressed in this article are solely those of the authors and do not necessarily represent those of their affiliated organizations, or those of the publisher, the editors and the reviewers. Any product that may be evaluated in this article, or claim that may be made by its manufacturer, is not guaranteed or endorsed by the publisher.

## Footnotes

^1^ICD10 codes: J01.-; J02.-; J03.-; J04.-; J05.-; J06.-; J12.-; J18.-; J20.-; J21.-; and J22.-.

^2^ https://www.dzs.hr/hrv/publication/StatisticsInLine.htm
